# Povidone-iodine Irrigation - A Possible Alternative To Lead Extraction

**Published:** 2011-07-03

**Authors:** Rishi Puri, Peter J Psaltis, Adam J Nelson, Prashanthan Sanders, Glenn D Young

**Affiliations:** 1 Cardiovascular Research Centre, Royal Adelaide Hospital; 2 Cardiovascular Investigation Unit, Royal Adelaide Hospital; 3 Disciplines of Medicine and Physiology, University of Adelaide, Adelaide, Australia

**Keywords:** ICD/PM, device erosion, povidone-iodine

## Abstract

Pocket infection and erosion remain the commonest (class 1) indication for pacemaker (PM) or implantable cardiac defibrillator (ICD) lead extraction. However, tranvenous lead extraction is not without significant risk of serious complications, particularly in patients with chronically implanted leads or ICD leads specifically. The paucity of cardiologists adequately experienced to undertake this high-risk procedure also means that its availability is limited to relatively few specialist institutions, yet more conservative 'lead-preserving' treatment options have not been well-reported. We describe the first reported case of a chronically eroded and infected ICD generator, managed conservatively with 5-days of povidone-iodine closed irrigation, followed by re-implantation of a new ICD on the contralateral side. With satisfactory long-term follow-up, this successfully averted the need for lead extraction in our elderly patient. We advocate the need for formal prospective evaluation of conservative therapeutic strategies of PM and ICD pocket infections. Although not gold standard, it provides an important therapeutic alternative in resource-limited areas.

## Case report

**Figure 1:**
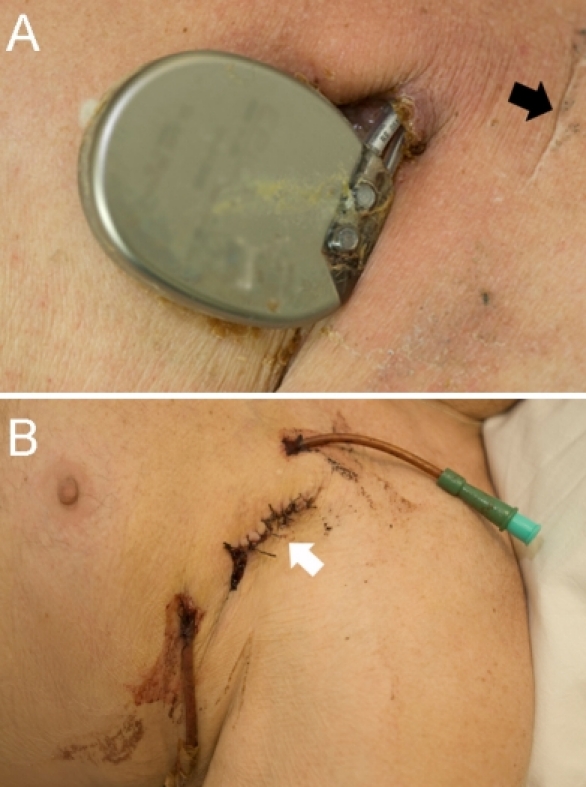
(A) Photograph of the extruded ICD generator, showing encrustation adjacent to the exposed leads. Black arrow indicates the incision scar from the original implantation procedure. (B) Photograph demonstrating the Z-incision (white arrow) created for the purpose of wound debridement and the irrigation (top)-drainage (bottom) tubes in situ for closed system povidone-iodine irrigation

A 79-year-old male with ischaemic cardiomyopathy (left ventricular ejection fraction 22%) had an ICD inserted in early 2002. In March 2007 he required a generator battery change, undertaken with pre- and peri-procedural antiobiotic cover. Seven months later, he noticed mild erythema and subcutaneous swelling over the generator. Despite treatment with oral flucloxacillin, the swelling increased in size over the following weeks and began to drain a purulent discharge, from which Morganella morganii and Enterobacter species bacteria were cultured. Although he remained afebrile, with normal leucocyte count and negative blood cultures, inflammatory markers were elevated (ESR 72mm/hr and CRP 87mg/L). After excluding concomitant valve or lead-related endocarditis, the patient was treated with a four-week course of intravenous ciprofloxacin and flucloxacillin, followed by a prolonged course of oral antibiotics. Whilst this resulted in normalisation of his inflammatory markers and notable improvement in the swelling over his infection site, there was ongoing pressure necrosis of the overlying skin and this led to the gradual extrusion of his ICD generator (Figure 1A). The preferred treatment strategy strongly recommended at this stage was for complete removal of both the generator and transvenous pacing leads, however, the patient declined this. Consequently, it was decided to attempt a novel 'lead-preserving' strategy as a 'bail-out' alternative, yet conservative option.

**Figure 2:**
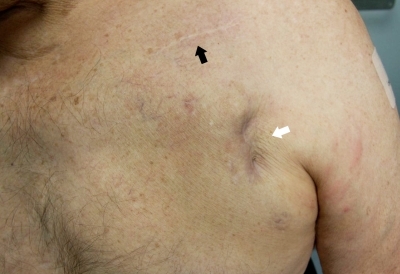
Photograph at 24-month follow-up, at which time the patient was asymptomatic and had normal serum inflammatory markers. Both the original implantation scar (black arrow) and the debridement incision scar (white arrow) are evident. Although the latter is associated with skin indentation, palpation of the adjacent area did not indicate evidence of ongoing infection.

After premedication with intravenous vancomycin, a Z-incision was made over the extruded generator, and it was removed. The lead was disconnected from the battery, capped, and allowed to remain in situ. Necrotic tissue was extensively debrided, and 2 drains were inserted (an entrance and exit drain) and sutured at both ends of the Z-incision, thus creating a closed irrigation system (Figure 1B). A 50mL solution of diluted povidone-iodine [5mL povidone-iodine (1% w/v) with 45 mL normal saline] was infused via the drains into the wound, four times each day for one week. There was no specific flushing interval or infusion rate allowing the povidone-iodine to bathe and drain naturally from the exit drain.  During this time intravenous antibiotics and cardiac monitoring were continued. After 5-days, the drains were removed, the wound closed, and a new single lead ICD system was implanted on the contralateral side. The patient was discharged home on oral flucloxacillin and ciprofloxacin for a further two weeks. At 24-months there was no sign of wound dehiscence or breakdown and subclinical markers of infection (white cell count and CRP) were all normal (Figure 2).

## Discussion

Wound infection can be a devastating complication of PM/ICD device therapy, especially when it progresses to result in pressure necrosis and erosion of the overlying skin and ultimately extrusion of the device generator. In this setting, guidelines strongly advocate complete removal of all the components of the PM/ICD system, including the extraction of transvenous leads [1].  Although novel methods of lead extraction (laser or radiofrequency powered extraction sheaths) have resulted in published success rates in excess of 95%, potential complications (myocardial avulsion or vascular tear with subsequent tamponade, air embolism, septic shock and death) are significant [2]. Such risks depend greatly on operator experience and necessitate the on-site availability of prompt cardiac surgical back-up [1]. Therefore operator and site selection are crucial in optimizing patient outcomes from lead extraction surgery. The recently reported 95-98% success rates for PM/ICD lead extraction occurred in a single, high volume centre that attempted 975 endovascular lead extractions over a 7-year period [2]. Moreover, the need for laser assistance for extraction was more likely for ICD leads (OR 3.44, 95% CI 1.84-6.43) as well as for leads implanted longer than 3.4 years (OR 6.15, 95% CI 3.35-11.28) [2]. Removal failure rates of up to 20% have been previously reported for those leads implanted longer than 8- years, despite the availability of newer lead extraction techniques [1].

Although the current guidelines do not support the management of deep pocket infections without explantation of the all of the PM/ICD and related hardware, there have been reports of patients refusing the option of lead extraction, or considered excessively high risk for this procedure, who have been successfully treated more conservatively [3,4]. Most of these previous case reports and small case series have described a combination of systemic treatment with intravenous antibiotics, extensive local debridement and pocket modification/relocation, and open or closed irrigation with bactericidal antibiotic solutions. The rationale for this approach has been that as the materials encasing the device generator and electrodes are inert, careful surgical debridement and re-sterilization of the infected pocket and generator may eradicate the infection without the need for complete replacement of the entire PM/ICD system [3]. In one series of 19 patients with infected pacemaker pockets, all cases were managed by extensive pocket debridement and enlargement, followed by 5-days of closed saline-antibiotic irrigation, without any instances of re-infection after a mean follow-up period of 22-months [5]. More recently another group reported on two lead-preserving procedures for PM pocket infection [4].  One of these procedures preserved the full length of the lead, whilst the other, the distal portion of the lead. After wide pocket debridement, saline solution irrigation, re-scrubbing and redraping, the pocket and lead were disinfected with povidone-iodine soaked gauze for 15 minutes.  The lead was then re-tunneled, with a new PM generator implanted in a new pocket. This method led to successful PM preservation out to a mean of 58-months follow-up [4].

The application of closed irrigation systems for the treatment of infected and extruded cardiac pulse generators has seen a variety of irrigation solutions used, including diluted cephalothin, and antimicrobial mixtures containing neomycin/bacitracin/polymyxin and tyloxapol/tobramycin [5]. Our case is the first to describe the successful use of a 5-day closed povidone-iodine irrigation regimen, obviating the need for high-risk extraction of a 6-year old implanted ICD lead.

Although early in vitro experiments, coupled with mixed results from clinical trials suggested antiseptics may impair wound healing, the emergence of bacterial resistance to antibiotics has resulted in a reappraisal in the use of antiseptics; particularly the use of iodine compounds [6]. Povidone-iodine exhibits strong and immediate bactericidal properties covering a wide range of micro-organisms, without the acquisition of resistance with its long-term use [6]. In addition, iodine has also been found to be effective against controlling MRSA outbreaks [6]. Trials using povidone-iodine irrigation for traumatic wounds have shown lower infection rates compared to placebo, although there are reports of a small risk of iodine induced thyroid disease and hypersensitivity reactions warranting diligence with its use [6].

Although conventional surgical practice recommends the removal of all foreign bodies in the presence of infection, our case demonstrates intermediate to long-term success with a conservative approach in a patient who refused complete removal of his infected, extruded system. The key components of management involved thorough surgical debridement and removal of the capsule, adequate pocket enlargement to ensure wound closure without tension, and proper positioning of a closed irrigation system for a 5-day period. We propose that a feasible, cost-effective, low-risk, 'lead-preserving' strategy could be considered in select patients with PM/ICD infection and generator extrusion, who do not have access to operators/institutions well experienced in transvenous lead extraction as may be the case in resource-limited areas, or whose co-morbidities preclude aggressive surgical management. This may particularly suitable in patients colonized with less virulent organisms. Given the relative lack of access to expertise within centers of excellence in PM/ICD generator removal, we advocate a formal, prospective evaluation of such treatment in patients refusing or unable to have the current gold-standard of treatment.

## References

[R1] Smith MC (2008). Extraction of transvenous pacing and ICD leads. Pacing Clin Electrophysiol.

[R2] Jones SO (2008). Large, single centre, single-operator experience with transvenous lead extraction: outcomes and changing indications. Heart Rhythm.

[R3] Har-Shai Y (1990). The management of exposed cardiac pacemaker pulse generator and electrode using restricted local surgical interventions; subcapsular relocation and vertical-to-horizontal bow transposition techniques. Br J Plast Surg.

[R4] Yamada M (2002). Surgical lead preserving procedures for pacemaker pocket infection. Ann Thorac Surg.

[R5] Hurst LN (1986). The salvage of infected cardiac pacemaker pockets using a closed irrigation system. Pacing Clin Electrophysiol.

[R6] Khan MN (2006). Antiseptics, iodine, povidone iodine and traumatic wound cleansing. J Tissue Viability.

